# Usability Testing and the System Usability Scale Effectiveness Assessment on Different Sensing Devices of Prototype and Live Web System Counterpart

**DOI:** 10.3390/s26020679

**Published:** 2026-01-20

**Authors:** Josip Lorincz, Katarina Barišić, Vjeran Vlahović

**Affiliations:** 1 Faculty of Electrical Engineering, Mechanical Engineering and Naval Architecture (FESB), University of Split, Ruđera Boškovića 32, 21000 Split, Croatia; 2 Croatian Academy of Engineering (HATZ), Kačićeva 28, 10000 Zagreb, Croatia; 3 Netgen d.o.o., Savska Cesta 182, 10000 Zagreb, Croatia; katarina@netgen.io (K.B.); vjeran@netgen.io (V.V.)

**Keywords:** human–computer interaction, user experience (UX), system usability scale, accessibility, sensing, mobile device, testing, prototype, live web, Figma, development

## Abstract

During the process of digital-system development from prototype to live implementation, differences in user interactions, perceived usability, and overall satisfaction can emerge. These differences often arise due to various factors, which may include the fidelity of the software prototype, the limitations of the prototyping tool, and the complexity of the live digital system. Recognizing these potential usability discrepancies between prototypes and live digital systems, assessment of how well user experience (UX) test approaches, such as usability testing and the System Usability Scale (SUS), reflect the UX in using the digital-system prototype and its counterpart deployed live system emerged as an important research gap. To address this gap, this study compares usability testing and SUS results among a Figma web prototype and its counterpart live web digital system, for the telecom service extension process as a representative digital-system case study. The research study involved a testing process with a total of 10 participants across the Figma prototype and live-web-system test environments, in which different sensing devices that included versatile types of mobile phones were utilized. The research study presents usability testing results related to the overlap in perceived usability issues for the same digital-product developments in both testing environments, which are experienced on different types of mobile sensing devices. The usability testing results are presented as reports on the frequency of occurrence of web system usability issues and corresponding severity levels. The obtained results demonstrated that prototype testing is highly effective for detecting a wide range of usability issues early in the digital-product development phase. The paper also evaluates the predictive capabilities of SUS assessment for the case of the Figma web prototype and its counterpart live web system in the phase of digital-product development. The results show that the SUS evaluation, when applied to digital-system prototype testing, can provide early in the development process a reliable indication of the perceived usability of its counterpart digital system, once it is developed and deployed. The findings presented in the paper offer valuable guidance for software designers and developers seeking to make prototypes and their counterpart real digital-product deployments with improved digital-product overall user experience.

## 1. Introduction

In the early 1980s, the field of human–computer interaction (HCI) emerged with a focus on assessing system usability, which emphasizes the design of systems that are efficient, easy to use, and supportive of users in achieving their goals. This foundation later expanded into the broader concept of user experience (UX), which also considers emotional, aesthetic, and contextual factors in assessing the UX of digital products [1]. Since the early days of HCI, usability testing and the System Usability Scale (SUS), as two well-established industry UX-testing methods, have been widely adopted to assess the usability of digital systems. Today, they remain the most relevant approaches in evaluating digital-system usability, thus providing insights into how users interact with digital systems, such as websites, web and mobile applications, software platforms, e-commerce systems, etc. [2,3].

Usability testing as a standalone method and the SUS as an independent questionnaire offer distinct contributions to UX evaluation of digital systems. While usability testing uncovers specific usability issues, the SUS captures overall impressions of usability. When used in combination, they allow researchers to balance qualitative testing insights with quantitative test data, thus providing a more robust understanding of the UX in using digital systems.

Moreover, in the rapidly evolving landscape of digital-product design, software tools like Figma have revolutionized the way prototypes of interactive digital systems are created and tested [4]. These prototypes closely mimic the final system, allowing for early detection of usability issues before implementation. However, as digital-systems development progresses from prototype to live implementation, differences in user interactions, perceived usability, and overall satisfaction can emerge. These differences often arise due to various factors such as the fidelity of the software prototype, the limitations of the prototyping tool, and the complexity of the live digital system. Prototypes created in software tools like Figma, while valuable, may not fully capture the nuances of the live digital system, as they are typically streamlined and focused on core interactions and specific user journey flows. Prototypes serve as models of digital systems, reflecting the goals defined during the research and planning phase of digital-system development [5]. They are designed to understand and validate, early in the development process, key aspects of digital-product functionality and UX. However, the live system often involves a broader range of interactions and issues that may not arise in the counterpart prototype system. These may include factors like slow backend responses, real-time data processing, software bugs, and variations in user-specific content. Such factors can present unexpected challenges that may not be apparent during prototype testing.

Recognizing potential UX discrepancies in using prototypes and live digital systems, it becomes essential to evaluate how well usability testing and the SUS analyses performed on a prototyped digital system reflect the UX of using the counterpart live digital system. To address this gap, this study compares usability test outcomes for a prototyped web system developed in the Figma software tool and its counterpart live web system. In both (Figma and live web) test environments, the execution of user tasks in the web system and the user journey were identical and were based on a typical telecom operator user service extension flow. In this user journey, existing customers extend contractual obligations for fixed-network data and television services via a web-based online platform, over their own sensing devices that include versatile mobile phones with different software versions and operating systems.

Thus, the motivation behind this research is to provide an analysis of the differences in user interactions, observed usability, and overall satisfaction perceived with different mobile sensing devices for the execution of user tasks in the prototyped web system and its live web system counterpart. By understanding these differences, this paper highlights the strengths and limitations of each test environment, ultimately giving guidance to UX practitioners in making informed decisions during the prototype and live digital-system design and development process.

Therefore, this paper contains the following main contributions. First, the paper systematically analyzes the overlap among usability issues characteristic of two product development phases of the same digital system, which are a Figma web system prototype development phase and its live-web-system counterpart. The analysis of the usability issue overlap is performed based on reports on the occurrence of UX issue frequency and severity levels, enabling a comparative usability assessment of the Figma web prototype and live web systems in their design phase. Second, by means of SUS evaluation, the paper benchmarks perceived usability in the context of both the Figma web prototype and live-web-system test environments. The perceived usability analyses also report dispersion statistics of SUS results based on standard deviations and 95% confidence intervals, thus enabling comparisons of the predictive capabilities of SUS evaluations for the Figma web prototype and live web system. Third, the paper provides a Croatian translation of the SUS questionnaire items to facilitate standardized perceived usability measurement in Croatian practice and research.

The rest of the paper is organized as follows: a review of related work on digital-system usability testing and the SUS evaluation is presented in Section 2. In order to contextualize the research presented in this work within the theoretical background, in Section 3, the theoretical fundamentals of usability testing, the SUS, and the Figma prototype tool are described. The research methodology based on usability testing and SUS evaluation is described in Section 4. Section 5 describes the usability and SUS test scenarios executed for the live web system and its Figma prototype. In Section 6, the results of the performed usability tests and the SUS evaluations with the obtained statistical results of the SUS evaluation are presented and discussed. Concluding remarks and a description of future research activities are provided in the last Section 6.

## 2. Related Work

Although usability testing represents a broad term that can span from testing the usability of different software platforms [6] or standalone hardware systems [7] in different usability cases, usability testing has mostly become common practice for testing partial or complete software products. More specifically, usability testing and the SUS have been extensively studied and validated in various contexts of the design and development of software products.

Usability testing is a broadly accepted approach in assessing whether users can achieve their goals during interaction with a digital system [8,9]. Within Rohrer’s classification of user experience research methods, usability testing is positioned as a qualitative and behavioral method, with the primary aim of observing how users interact with a product to uncover usability issues [3,10].

In addition, the foundational work on the SUS presented in [11] introduces the SUS as a quick and reliable tool for assessing system usability. The early evidence of SUS statistical sensitivity has been provided in [12], where it was shown that of five methods for assessing usability satisfaction, the SUS method was the quickest to converge towards the final “correct” conclusion. These findings highlight the advantage of using the SUS for evaluating usability satisfaction, particularly in situations where the number of participants is limited [12]. A similar study in [8] also emphasized that collecting multiple metrics in usability testing is advantageous, as it provides a better picture of the overall user experience than any single measure can.

While the SUS was originally intended as a usability metric, recent studies increasingly use it to assess new domains related to the broader UX. Therefore, the SUS has been applied not only to evaluate digital-product effectiveness and efficiency, but also satisfaction, loyalty, and perceived digital-product quality [9]. In the contexts of mobile health and education, high SUS scores have been shown to correlate with Net Promoter Score (NPS), indicating alignment with user loyalty and overall satisfaction [9]. Additionally, the work [13] demonstrated moderate correlations between SUS scores and perceived aesthetics score (r ≈ 0.5), as well as with positive emotional valence score (r ≈ 0.35), which suggests that the SUS can reflect some affective reactions of participants involved in testing.

Additionally, some recent studies have started to introduce usability and SUS examination of digital-system prototypes. In [14], a prototype digital system was developed and tested with 10 test participants using usability testing and the SUS questionnaire. In [15], the prototype electronic-personal health record platform was evaluated with the SUS across different user contexts. Furthermore, the SUS was used to assess a prototype for integrating laboratory visualizations across a number of user tests in [16]. In [17], the prototype of a clinical decision support digital system was assessed using the remote think-aloud method and SUS evaluation.

However, this previous research on the topic of usability testing of digital products shows a lack of research related to comparing usability testing and SUS results on real, operating digital systems and their equivalent prototypes. In summary, the presented related work focuses on single-phase evaluations, typically on live digital systems, rather than on comparing the same user journey across multiple phases of digital-product development (e.g., from the prototype pilot phase to live digital-system deployment phase). This paper fills this gap by presenting the results of analyses that compare usability testing outcomes and SUS scores on the Figma web system prototype and its counterpart live web systems. Unlike prior research work, this paper examines the same digital system developed as the Figma web prototype and live web implementation, and the same user journey, to compare the results of the two UX test approaches. For such a user journey, the paper also quantifies usability issue overlap based on issues’ severity and frequency of occurrence, and compares SUS results across both the Figma web prototype and live-web-system test environments. Therefore, these analyses assess whether usability issues of different severities can be detected during the design phase of the digital-product prototype, and whether SUS questionnaire results obtained during the digital-system prototype development phase predict SUS results of the counterpart digital system in the live implementation phase. Thus, this paper aims to contribute to the ongoing discourse on effective UX evaluation methods, offering insights that can enhance the design and development of user-centric digital systems.

## 3. Theoretical and Conceptual Foundations of the Study

This section presents the theoretical and conceptual foundations of the performed research study. More specifically, in this section, the fundamentals of usability testing and the SUS are presented with the description of specifics related to each of the usability testing concept implementations. Also, the description of the Figma digital-system prototyping software tool used for the development of a web prototype is given.

### 3.1. Usability Testing

**Figure 1:**
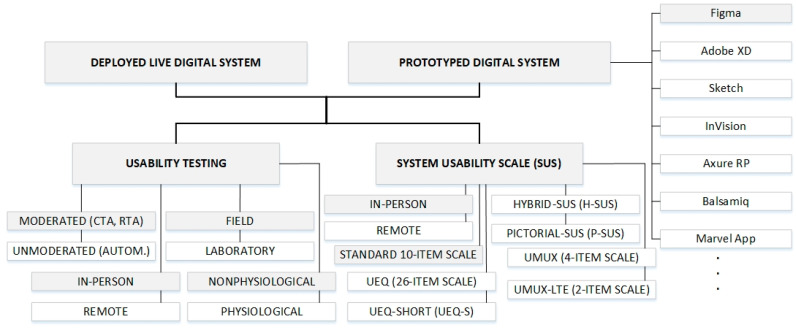
Classification of usability test approaches on different test environments (shaded indicates test approaches and test environments used in analyses).

Figure 1 presents a classification of usability test approaches on different test environments, with shaded regions indicating test approaches and test environments used in analyses in this work. According to Figure 1, usability testing is one of two UX testing types used in this work for assessing the perceived usability of the Figma web prototype and its counterpart live web system. Usability testing involves direct observation of users as they complete tasks within a system, identifying areas of friction, and gathering qualitative data on user performance and satisfaction. Usability testing is particularly effective for identifying specific interaction issues, validating design assumptions, and uncovering patterns in user behavior. This testing method has been widely accepted as a cornerstone of UX research since the late 1980s and has a critical role in supporting the iterative digital-product design process by ensuring continuous improvement and refinement of user-centric digital systems. These advantages of usability testing are the main reasons for selecting usability testing as an approach for assessing the perceived usability of the analyzed web system in the research presented in this work (Figure 1).

Usability testing methods can be categorized based on different classifications (Figure 1). With respect to context, one can distinguish between laboratory-based testing, which provides a controlled environment to minimize distractions, and field testing, which enhances ecological validity by capturing user interactions in their naturalistic settings [3,18]. In the analyses presented in this work, the usability testing was performed as field testing (Figure 1).

Furthermore, usability testing can be classified as moderated or unmoderated (Figure 1). Moderated sessions involve a facilitator who guides participants and may ask probing questions, either concurrently or retrospectively. Concurrent think-aloud (CTA) protocols involve verbalizing thoughts during task execution (Figure 1), while retrospective think-aloud (RTA) sessions elicit user reflections post-task, often with video replay [3,19]. In contrast, unmoderated testing relies on automated platforms to capture user behavior without real-time facilitation, which may limit the depth of insights, but enhances scalability and geographic reach. In the analyses presented in this work, the usability testing was performed as moderated testing sessions (Figure 1).

Remote and in-person usability testing represent another area of usability testing categorization (Figure 1). It can be synchronous (e.g., remote moderated testing via video conferencing) or asynchronous (e.g., unmoderated tests using specialized tools). Although some nuances may be lost in remote settings, several studies suggest that both remote and in-person testing identify similar categories of usability issues [3,18]. In the analyses presented in this work, the usability testing was performed as in-person usability testing (Figure 1).

In addition to behavioral (nonphysiological) observations and performance metrics that represent another category of usability testing, some studies increasingly incorporate another usability testing category that is based on physiological data. This type of usability testing includes eye-tracking, skin conductance, and heart rate variability to gain deeper insight into user behavior and affective responses [19]. These measures offer additional insight into cognitive load, emotional arousal, and attention distribution, supplementing traditional usability metrics [19]. In the analyses presented in this work, the behavioral (nonphysiological) data have been included in usability test results (Figure 1).

There is no consensus in the relevant literature regarding the recommended number of participants for usability testing [20]. Several separate studies independently provided evidence supporting the effectiveness of usability tests conducted on a small sample of participants. Authors in [21] demonstrated that the optimal cost–benefit ratio in usability testing is achieved with three to five participants, as testing with this sample size uncovers approximately 85% of usability issues. Similarly, refs. [22,23] reported that small studies are capable of identifying 80–85% of usability issues within a given test scenario. However, other empirical work challenges this view by showing high variability in problem discovery depending on context and participant characteristics [24]. In addition, some studies emphasize that the selection of appropriate test participants and the quality and coverage of tasks are more critical to identifying usability problems than the absolute number of users participating in tests [19].

Nevertheless, both refs. [25,26] underscore that the appropriate number of users participating in tests should be determined based on the context and goals of the usability study. Specifically, in [26] it is argued that a sample of five test participants is sufficient in the majority of qualitative usability tests, particularly when the same system is evaluated iteratively. This is because each successive round of testing uncovers new insights, while maintaining a high return on investment. In [25], this conclusion is further supported by highlighting that iterative testing allows for incremental improvements across testing cycles, which reduces the need to identify every possible issue in a single round of the testing cycle. For that reason, in the analyses presented in this work, the usability testing was performed on five test participants.

### 3.2. System Usability Scale Assessment

The SUS is the second of the two UX testing types used in this work for assessing the perceived usability of the Figma web prototype and its counterpart live web system (Figure 1). Among the standardized instruments for usability testing, the SUS is the most widely adopted in practice [19]. The SUS, as a usability testing method, was developed by John Brooke in 1986 and published in 1996 [11]. The SUS is a ten-item Likert scale questionnaire used to evaluate the perceived usability of a system. The SUS provides a quantitative measure that complements the qualitative insights from usability testing, offering a holistic view of user experience. It can be integrated into standard task-based usability testing, enhancing the system usability assessment by quantifying user satisfaction and perceived usability [27].

As an indicator of SUS reliability, the 10-item SUS questionnaire has demonstrated high internal consistency, with a reported Cronbach’s alpha internal consistency measure of 0.91, which was obtained on over 2300 SUS questionnaires collected across more than 200 usability studies [2]. This strong reliability, alongside its simplicity and robustness, has made the SUS a widely adopted tool in UX evaluations across various industries and contexts. Recent work [28] reaffirmed the reliability of the SUS as a test method by reporting a high level of internal consistency (Cronbach’s alpha of 0.92) in contemporary usability evaluation of digital systems. These characteristics of the SUS are the main reason for selecting the SUS as an approach for assessing the perceived usability of the Figma web prototype and its counterpart live web system in the research presented in this work (Figure 1).

The analyses of SUS statistical sensitivity presented in [12] indicate that the SUS 10-item questionnaire reaches 75% agreement at a sample size (number of test participants) equal to eight, and 100% agreement with a sample size of twelve test participants. In the analyses presented in this work, the SUS testing was performed as in-person usability testing and completed by test participants immediately after the usability testing task (Figure 1).

The widespread adoption of the SUS can be partially attributed to the availability of linguistically validated versions in a wide range of target languages, including Arabic, Chinese, French, German, Hindi, Italian, Persian, Polish, Portuguese, Slovene, and Spanish [13]. The Slovene SUS version (SUS-SI) [29] is particularly relevant in the context of this study due to the linguistic and cultural proximity between the Slovene and Croatian languages. Thus, the Slovene version of the SUS questionnaire offers a useful reference point for the Croatian translation of the SUS questionnaire, which is developed and presented in this work.

Several adaptations of the SUS have also been developed to enhance the user experience of the questionnaire itself. The Hybrid System Usability Scale (H-SUS) integrates verbal and pictorial elements to improve respondent motivation and engagement (Figure 1), while maintaining psychometric comparability with the original SUS [13]. The additional Pictorial System Usability Scale (P-SUS) approach provides a fully pictorial version of the SUS questionnaire to support accessibility for children or low-literacy populations (Figure 1) [25].

To assess broader aspects of user experience beyond usability, another SUS-based testing method known as the User Experience Questionnaire (UEQ) was developed (Figure 1) [30]. It includes 26 semantic differential items grouped into six subscales: attractiveness, perspicuity, efficiency, dependability, stimulation, and novelty. Its short form version UEQ-Short (UEQ-S), retains psychometric robustness while reducing respondent burden (Figure 1) [15]. UEQ is particularly useful in distinguishing between pragmatic and hedonic aspects of user experience.

In response to the need for shorter formats of the SUS questionnaire, ref. [31] introduced the Usability Metric for User Experience (UMUX), which represents a four-item scale that was developed based on International Organization for Standardization (ISO) 9241-11 [32] criteria (Figure 1). Its abbreviated version, UMUX-LITE, consists of only two items and has been shown to correlate highly with SUS (Figure 1) [33], offering a practical alternative to SUS in time-constrained settings. Despite their brevity, both UMUX and UMUX-LITE exhibit adequate psychometric properties and are particularly suited for benchmarking usability in large-scale or unmoderated studies [8]. Since the analyses presented in this work were based on moderated usability testing, the standard 10-item SUS questionnaire has been used for assessing the perceived usability of the Figma web prototype and its counterpart live web system (Figure 1).

### 3.3. Figma Digital System Prototyping Software Tool

In the market, different software tools like Figma, Adobe XD, Sketch, InVision, Axure RP, Balsamiq, Marvel app, etc., can be used for performing prototyping of digital systems (Figure 1). The Figma mobile application version 24.15.0 for iOS and Android was used for the development of the web system prototype in the analyses presented in this work. The Figma application is more than just a digital design tool used for creating user interfaces of websites, mobile applications, or other digital products. It represents a cloud-based platform for prototyping, collaboration, and interactive design of digital systems. Figma supports real-time teamworking, which enables designers, developers, and UX experts to conduct usability testing even during the design phase of the digital system development process.

The Figma digital tool facilitates rapid prototyping of user interfaces and enables simulation interactions and user flows, which help validate design concepts early in the development of digital systems. Figma’s sharing features allow prototypes to be presented to users on their own sensing devices, independently of whether they are using a desktop or mobile device. This approach is particularly valuable because it enables testing on the same sensing devices that users would typically use to interact with the digital system. Figma supports the evaluation of key aspects of user experience, including intuitivity of using the digital product, efficiency of task completion, the identification of common errors encountered during interaction of the user with the digital product, and overall user satisfaction with the digital-product design [5]. For these reasons, the Figma software tool was selected as the platform for developing the web system prototype.

## 4. Research Methodology

The study presented in this work employed a combination of usability testing and the SUS questionnaire to assess and compare the usability of a web system prototyped in Figma and its counterpart live web system. During the testing process, test participants were assigned to two distinct test environments, defined as the live web system and its counterpart Figma prototype. Table 1 provides an overview of how participants were distributed across different test environments and testing modes. In each test environment, five participants took part in attended usability testing, where test sessions were moderated and conducted in person.

The same testing criteria were used for all test participants, which included tests on the Figma prototype and live web system. According to Table 1, usability testing and the SUS have not been performed with the same test participants for testing the Figma prototype and live web system. The reason is a consequence of the fact that test participants after the first test will be familiar with the digital system due to testing memorability, and testing on the counterpart system will have no value (because the participants will know what to expect in every next step of testing).

### 4.1. SUS Testing Approach

For Figma web prototype testing, five test participants were involved in the SUS assessment. Although a larger sample of test participants (typically 10–15) is recommended to obtain highly stable SUS scores [12], a smaller number of test participants can still provide meaningful and valid insights, particularly in early-stage prototype evaluations. The SUS approach was shown to be robust even under small-sample conditions, and prior research demonstrates that the SUS can produce reliable usability assessments with as few as five participants. For example, Brooke, the original author of the SUS, in [11], noted that the SUS was intentionally designed to be “reliable even with small sample sizes,” making it suitable for formative testing of prototypes. Furthermore, studies presented in [12] found that samples of 4–6 participants can correctly identify relative usability differences between systems, especially when the objective is comparative rather than absolute measurement. Since the purpose of SUS testing in this study was not to estimate population-level usability with narrow confidence intervals, but rather to compare a prototype with its live-system counterpart, a smaller group of only five test participants is engaged in the Figma web prototype testing (Table 1). For that reason, this approach is methodologically acceptable for the prototype testing. Also, selecting the five test participants for Figma web prototype SUS testing is consistent with the recommended usability practice presented by Nielsen in [21], emphasizing that early-stage design evaluations (as is the one presented in this work) frequently rely on small samples to detect major usability trends. Therefore, the five test participants’ sample size used for the Figma web prototype’s SUS evaluation is adequate for providing a comparison with the live-web-system usability testing.

### 4.2. Usability Testing Approach

In the study, usability testing was conducted with a small sample of participants using a moderated concurrent think-aloud (CTA) protocol, in which a facilitator guided participants as they verbalized their thoughts during task execution. All testing sessions were conducted in person, ensuring direct observation of interactions and non-verbal expressions of users participating in testing. The goal of usability testing was to uncover key usability issues related to telecom operator service extension through the testing of the web system prototyped in Figma and its counterpart live web system. Such performed field testing provides insight into real-world usage conditions, which can include, for example, the impact of the work environment, the lighting of surroundings, or different user distractions [18].

Since usability issues are specific problems or obstacles encountered while interacting with a software system, such usability issues have an impact on the overall user experience, satisfaction, and effectiveness of the tested system usage. Thus, the focus of usability testing performed in this work was directed to achieving the main goals of usability testing, which are usability discovery and resolution [9]. The two separate groups of 5 participants were involved in usability testing, with one group testing the Figma prototype and the other testing the live web system (Table 1). This resulted in a total of 10 test participants distributed across two test environments. The participants performing the testing were all long-term users of the telecom company’s services, for whom the use of these services was known. The rationale for selecting five participants for performing usability testing (Table 1) is based not only on the relevant studies [21,22,23], which suggest that this sample size can uncover approximately 85% of usability issues, but also on the specific context of the study presented in this work. The tested system is subject to regular usability testing and continuous iterative refinement, which reduces the likelihood of major unresolved issues persisting between testing cycles [25,26]. Also, feedback from participants was analyzed in a way that identical issues reported by different participants were labeled as the same usability issues. This approach allows for the calculation of a usability issue’s frequency of occurrence for all conducted tests.

### 4.3. SUS Evaluation Methodology

Another method used for performing the usability analyses in this work was the SUS. Table 2 presents the items of the SUS questionnaire and their translation into the Slovenian and Croatian languages. As shown in Table 2, the SUS questionnaire consists of ten statements rated by respondents on a Likert scale, ranging from 1 (strongly disagree) to 5 (strongly agree). The SUS mandates that all questionnaire statements should be rated. If respondents are unsure about some questionnaire statement, they should mark the center point at the middle of the scale equal to 3 [11].

For odd-numbered, positively worded questionnaire statements indicated in Table 2, a value of one is subtracted from the respondent’s score. For instance, if a score of 3 is given for such an odd-numbered, positively worded questionnaire statement, the transformed score becomes equal to two (2 = 3 − 1). For an even-numbered, negatively worded questionnaire statement indicated in Table 2, the respondent’s score is subtracted from a value equal to five. For example, if a respondent’s score for such an even-numbered, negatively worded questionnaire statement is equal to four, the transformed score becomes equal to one (1 = 5 − 4).

After transforming all questionnaire statement (item) scores, the total SUS score is summed and multiplied by 2.5 to yield the final SUS score on a 0–100 scale, with higher scores indicating higher perceived usability [11]. A perfect score is achieved when all positively worded items receive a 5 and all negatively worded items a 1. This transformation enhances the clarity and interpretability of results, ensuring that on the global level, project managers, product managers, and engineers can quickly grasp the outcomes without the need for additional explanation [34].

In practice, the SUS questionnaire is administered after users interact with a system, but before any discussion on system usability, and thus, the SUS captures immediate reactions from participants in testing. In this study, the SUS score was obtained for two groups of participants. One group of 5 participants completed the SUS after usability testing of the Figma web system prototype, while another group of 5 participants completed the SUS after testing the live web system (Table 1). All 10 test participants were existing telecom operator service users.

#### 4.3.1. SUS Questionnaire Score Statistics

In the analyses presented in this paper, the SUS scores obtained based on the performed SUS evaluations are statistically assessed. The overall perceived usability, represented by the average SUS score for the system, is calculated as the mean of all individual respondents’ SUS scores. The mean value (m) represents the average score value of all SUS questionnaire responses. It is calculated by summing all individual SUS questionnaire scores (x_i_) and dividing by the total number of SUS questionnaire responders (N) as follows:

(1) $$m=\frac{\sum_{i=1}^{N}x_{i}}{N}$$

where i = 1, …, N represents an index of each responder.

The sample standard deviation (SD) quantifies the amount of variation or dispersion in the SUS questionnaire scores and is expressed as follows:

(2) $$SD=\sqrt{\frac{1}{N-1}\sum_{i=1}^{N}{\left(x_{i}-m\right)}^{2}}$$

The 95% confidence interval (CI 95%) is calculated according to the following relation:

(3) $$CI\;95\%=m\pm 1.96\frac{SD}{\sqrt{N}}\;$$

The CI 95% provides an interval range within which the confidence that the true mean SUS score lies is equal to 95%.

### 4.4. Croatian Translation of the SUS Questionnaire

To ensure its global applicability, the original SUS questionnaire has been translated and validated in multiple languages and cultural contexts [27]. An example of the SUS questionnaire in the English and Slovenian languages is presented in Table 2. However, interpreting questionnaire items (statements) from the original English language in different languages can lead to variations in the understanding of questionnaire items (statements). Additionally, a direct translation of items (statements) performed using automatic translators from the English language to a specific language may not perform equivalently, due to linguistic and cultural differences in the understanding of translated questionnaire items (statements). Thus, validation of the translation of SUS questionnaire statements in a specific language is essential for implementing an accurate SUS questionnaire.

Since there was no previously published translation of the SUS questionnaire in the Croatian language, which is the national language of the authors of this paper, Table 2 presents translated SUS questionnaire items (statements) in the Croatian language. The translation was created by combining items (statements) from the original English language questionnaire and the Slovenian language psychometrically validated questionnaire translation presented in [29]. Besides the English language, the validated SUS questionnaire in the Slovenian language was used since the Slovenian and Croatian languages have similarities, and those similarities help in obtaining a translation that is in line with the linguistic and cultural understanding of translated questionnaire items (statements). According to the authors’ knowledge, this translation is the first published translation of the SUS questionnaire items (statements) in the Croatian language.

## 5. Test Scenarios

**Figure 2:**
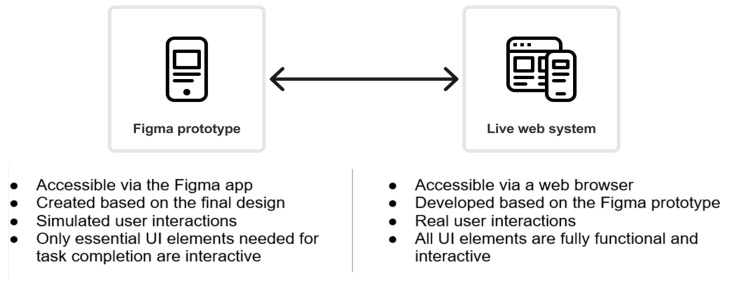
Overview of the key differences between the tested environments used in the analysis.

The analyses presented in this paper are based on conducting two independent usability tests. The first test was performed on a live web system, while the second one was performed on its counterpart prototype web system developed in Figma. The live web digital system and its equivalent Figma prototype were tested for a publicly available service on one prominent Croatian telecom operator website. Figure 2 outlines the main differences in characteristics of both test systems related to accessing and interacting within the test environments. Both test environments represent the same user journey, which involves the online extension of a contract for an already-active telecom operator service. Such a user journey represents the real-world situation that existing users of the telecom operator often encounter. Thus, participants in usability testing perform the same user journey that is tested on different mobile sensing devices for a web system prototyped in Figma and its counterpart live web system (Figure 2).

Considering the objective of this study, it was crucial to ensure comparable results of the conducted usability tests. A detailed test scenario was prepared to guide the usability testing process. The scenario consists of two main parts: an introduction to the usability testing for the test participant and a list of key steps and critical points that need to be followed for the moderator of the usability testing.

The introduction to the usability testing provides the test participants with the essential information about the purpose and rules of the testing, as well as a clear description of the tasks they are required to complete during testing. At the beginning of each test session, the moderator gives this information to the participants, ensuring they understand the context and objectives of the usability test. This introduction serves as a starting point for the test participants’ user journey and helps establish a structured and consistent testing environment (Figure 2).

The second part, related to the list of key steps and critical points, acts as a processing guide for the moderator during the usability testing session. It helps in monitoring the execution of essential tasks and gathering valuable insights related to each step of the test scenario. This structured approach ensures that moderators of the usability testing remain focused on critical areas that need to be tested, and guarantees that all participants follow a uniform and comprehensive testing process.

### 5.1. Overview of Usability Testing Steps and Tasks in the Tested User Journey

**Figure 3:**
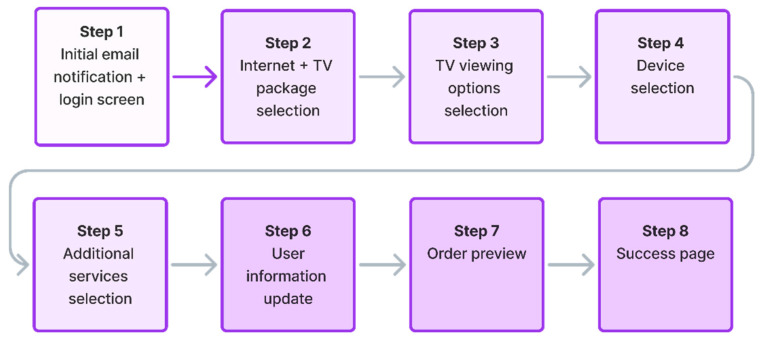
Overview of key steps in the tested user journey for telecom operator service extension.

To compare the results of usability testing and SUS scoring on a live web system and Figma web system prototype, a typical process flow on a telecom site was selected for testing purposes. The process flow is related to enabling the existing telecom operator customers with active services to extend their contractual obligation for fixed internet and television services via the web-based online platform. Access to the web-based online platform was provided to the test participants through web browsers on their mobile devices. Key steps in the tested user journey related to the telecom operator service extension are presented in Figure 3.

Prior to the execution of the test, a standardized briefing with test participants was conducted, in which participants were informed that the test evaluation is focused on the system’s interface rather than their personal abilities. To ensure consistency across all test sessions, test participants who were also actual telecom operator service subscribers were provided with a standardized scenario simulating the renewal of an expired internet and television (TV) contract with the specific objective of purchasing a promotional device. Also, during the briefing, demographic data regarding test participants’ occupation, age, and gender were collected.

**Figure 4:**
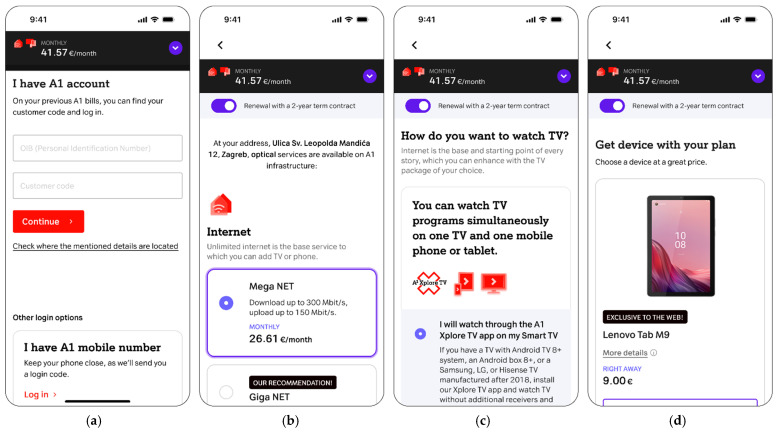
User interface sequence for Steps 1 to 4: (**a**) Initial email notification and login, (**b**) internet and TV package selection, (**c**) TV viewing mode selection, and (**d**) device selection.

**Figure 5:**
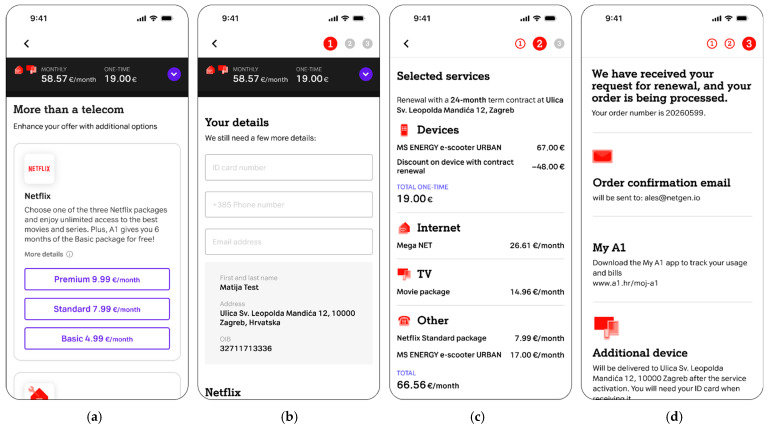
User interface sequence for Steps 5 to 8: (**a**) Additional services selection, (**b**) user information update, (**c**) order preview, and (**d**) success page.

This briefing ensures that all participants, regardless of the test environment (Figma prototype or live web system), approach the testing steps with the same user intent. The detailed user interface sequence iteration and the standardized moderator scripts for these steps in the tested user journey are presented in Figure 4 and Table 3 for Steps 1–4, and Figure 5 and Table 4 for Steps 5–8, respectively.

The tested user journey for telecom operator service extension, shown in to Figure 3, contains the following steps:

1. **Initial email notification and website login:** The user receives a telecom operator email with information related to the upcoming service contract expiration. The email communicates the option of extending the contract with the possibility of obtaining a service discount and an additional reward related to purchasing the offered devices at a discounted price. Following the email notification, the user is directed to log in to the telecom’s website (Figure 4a, Table 3).
2. **Internet package selection:** The user selects their desired internet speed from available options and chooses one or multiple TV packages according to their preferences (Figure 4b, Table 3).
3. **TV viewing options selection:** The user decides on their method of internet protocol TV (IPTV) watching (Figure 4c, Table 3).
4. **Device selection:** The user selects a promotional device (e.g., television, electric scooter, smartwatch, tablet, etc.) offered at a discounted price as part of the contract extension reward (Figure 4d, Table 3).
5. **Additional services selection:** The user has the option to upgrade their service with additional features such as streaming services, home insurance, etc. (Figure 5a, Table 4).
6. **User information update:** The user updates or completes their personal information, selects the method of receiving bills, and chooses how to activate any optional services selected in the previous steps (Figure 5b, Table 4).
7. **Order preview:** The user reviews a detailed summary of all selected services, prices, and applied discounts, and afterward selects the delivery address and payment method if purchasing devices (Figure 5c, Table 4).
8. **Success page:** The user receives an order confirmation and information on the next steps regarding service delivery and upgrades (Figure 5d, Table 4).

### 5.2. Selection of Test Participants

Table 5 gives general information about participants selected for system usability testing and their distribution among test groups. According to Table 5, the participants selected for usability testing were divided into two test groups (Figma prototype and live web), differing in age, gender, and educational background of participants. One test group tested the Figma web system prototype, and the other one tested the live web system (Table 5). The participants chosen are typical representatives of the current average users of the telecom operator services engaged in online service contracting. None of the selected usability testing participants were digital professionals, thus ensuring that the system usability testing involved average users with no deep technical familiarity with web-based systems.

Since the focus of usability testing was on the use case based on extending contractual obligations for the extension of telecom operator service, this use case had an impact on the participants’ selection criteria. Thus, the participant’s selection criteria were oriented towards the selection of participants who are well-acquainted with the telecom service brand’s communication and offerings. The participants were selected for usability testing based on their long-term usage of the telecom service, having an average contract duration of 11 years, and a minimum contract duration of 6 years (Table 5). The selection of participants with such durations of contracts for telecom service usage ensures the possibility of comparing users’ past experiences related to the process of contractual telecom service extension with those in the tested scenarios.

Also, from Table 5 can be noticed that each group of the five test participants has the same distribution of test participants in terms of age range. More specifically, there was one participant belonging to the 25–35 years old age range, one participant belonging to the 45–55 years old age range, and three participants belonging to the 36–44 years old age range. Therefore, the two groups have a completely homogeneous sample of test participants in terms of age ranges. Also, each test participant, regardless of age, has the same digital literacy and proficiency level (they use computers and digital tools in their daily work and have online shopping experience). Therefore, differences among 25-year-old and 55-year-old test participants do not contrast in terms of profile, since they have the same digital literacy and proficiency level. The selected groups of test participants are in line with Nielsen’s recommendation presented in [33], which confirms that a small sample of homogeneous test participants is enough for performing the tests on the Figma prototype and the live-web-system counterpart. Since the selected group of test participants is homogeneous in terms of digital literacy and proficiency level and in terms of having previous experience with the telecommunications service brand, the selected sample of the test participants, divided into two groups of five test participants, is appropriate for reliable testing.

### 5.3. Usability Testing and SUS Execution

**Figure 6:**
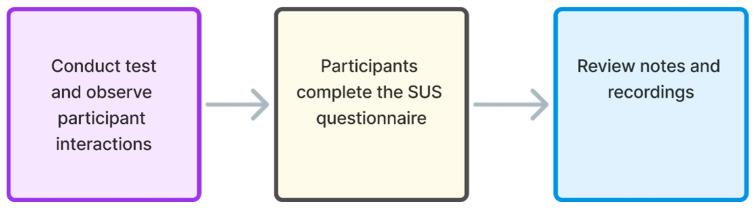
The three-step testing process.

The process of usability testing and SUS execution in this study is performed in three steps (phases). As shown in Figure 6, the three key steps of the testing process consist of conducting usability tests while observing participant interactions, completing the SUS questionnaire, and reviewing notes and recordings to document identified issues. In the first step (Figure 6), usability tests were conducted in person, employing field testing methods at locations that were chosen by the test participants and that represent locations where users spend part of their everyday time (at home, at work, etc.). Thus, the field testing was performed in controlled conditions, in the sense that noise or any other distractions were not present during testing, and tests were performed in spaces that are used by users as everyday living or working spaces, with which the test participants were familiar.

Each test session lasted between 40 and 45 min, and it was concluded following a moderated CTA protocol. This means that a moderator was physically present with the test participant and facilitated the test task while encouraging verbalization of thoughts during interaction. A test assistant, also co-located with the moderator at the test site, took structured notes and captured behavioral observations. Also, the same two examiners always led the testing (moderation) with the test participants and did the issue analysis report (in terms of recognizing and categorizing the issues). The same professional criteria were applied in terms of examiners determining whether some issue was categorised as a low-, medium-, or high-severity issue, and in this way, homogeneity was achieved. Thus, the data were collected based on the application of such a homogeneous approach.

The tests were performed based on ethical committee approval, and each test participant, before participating in the tests, signed their consent to participate in the tests. For screen recording, the Zoom mobile application was temporarily installed and used exclusively for recording screen activity and participant audio, without any remote communication.

Participants tested the live web system using their own mobile sensing devices (smartphones) running on either Android or iOS, while those who worked with the Figma prototype used the moderator’s iOS device to avoid installing the Figma application on their phones. The rationale for selecting mobile phones as a sensing device for performing usability testing is based on Google Analytics data collected for accessing the telecom website during the past 12 months. The analytical data shows that more than 70% of users of the telecom provider accessed the telecom provider’s website via mobile phones. Additionally, conducting all tests on mobile phones as sensing and interaction devices ensures uniformity in the testing approach, thereby eliminating potential perception differences that could arise from presenting test user interfaces on different types of user devices and screens.

To control variability in real user data and to ensure consistency in the available service options and pricing, participants during usability testing did not log in with their personal telecom operator service accounts. Instead, all test sessions were conducted using pre-configured test accounts that exposed the same user interface, service portfolio, and personalization-free content.

The second step in the testing process started after completing the usability test (Figure 6), during which each test participant filled out the SUS questionnaire for the system they had tested (either the Figma prototype or the live web system). Before starting to answer the SUS questionnaire, the moderator instructed participants involved in testing to respond without overthinking. However, in the case of uncertainty in answering a concrete SUS statement (item), test participants were instructed to select the middle option on the Likert scale (equal to three), which is also the approach recommended by the author of the SUS questionnaire [11]. To ensure consistent understanding of all statements, each SUS session was conducted as a moderated assessment using the think-aloud method, with participants being asked to verbalize their thoughts while answering.

The questionnaire was implemented using the Typeform platform [35]. The answering of the questionnaire was completed by test participants on their mobile phones immediately after usability testing, in the presence of both the moderator and assistant. SUS responses were then exported from Typeform to Google Sheets for further statistical analysis.

In the third step (Figure 6), the assistant reviewed the session notes and screen recordings to compile and systematize the identified usability issues. The overall testing was conducted over two days, with the same team (a moderator and a notetaker) leading each test session. This structured approach ensured consistency across all three processing steps and allowed for a detailed comparison of user interactions and feedback (testing results) obtained for testing of the Figma web system prototype and the live web system.

### 5.4. Data Analysis

**Figure 7:**
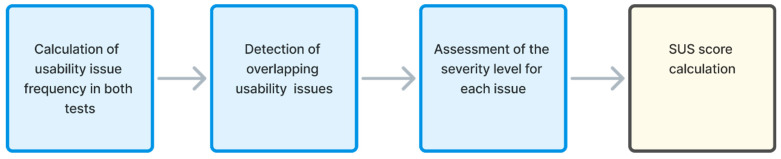
Phases of data analysis.

After conducting usability testing and SUS questionnaire test sessions, the data analysis of the findings was performed. The data analysis process presented in Figure 7 is divided into four phases, which include the calculation of usability issue frequency in both tests, the detection of overlapping usability issues, the assessment of the severity level for each issue, and the SUS score calculation.

#### 5.4.1. Calculation of Usability Issue Frequency in Both Tests

Usability issue frequency in both tests represents the frequency of occurrence of the same issue noticed by different test participants. The calculation was performed based on feedback from all test participants, which was analyzed in order to label identical issues reported by different test participants. This allows for the calculation of the usability issue’s frequency of occurrence in each conducted usability test.

#### 5.4.2. Detection of Overlapping Usability Issues

In the second data analysis phase, each detected usability issue was annotated by whether it was identified on the Figma web system prototype, live web system, or both. Also, the frequency of occurrence for each overlapping issue in each testing environment was analyzed. This information served as a basis for comparing the test results and obtaining conclusions in the subsequent processing phases.

#### 5.4.3. Assessment of the Severity Level for Each Issue

In addition to the detection of issue occurrence frequency or overlapping, an assessment of the severity level for each issue detected in usability testing was performed. The estimation of the severity levels was performed through labeling of usability issues, which are prioritized according to the judgment-driven assessment principle of the team members performing usability testing [21]. Table 6 provides illustrative examples from the tested telecom contract extension journey of some of the severity issues for different issue categories.

According to the evaluation and observations of the test team experts obtained during the testing process, the detected issues were categorized into the following three levels of severity:

1. **Low-severity usability issue** encompasses situations when a user may hesitate in selecting some options or pick the wrong option, which the user quickly resolves without incident (Table 6). Also, low-severity usability issues include situations when a user expresses minor irritation or annoyance regarding the usability of the tested system, but this does not affect the ability to complete tasks in the tested system.
2. **Medium-severity usability issue** encompasses situations when the user is frustrated or has difficulties in performing the task, but manages to complete it (Table 6). The medium-severity usability issue suggests that other users may be less tolerant of the inconvenience in test completion or that user frustration level can be high.
3. **High-severity usability issue** encompasses situations when a user cannot complete some or all tasks, or a user can complete the process but expresses extreme irritation with the process of task completion (Table 6). Also, the high-severity usability issue means that the user needs assistance to finalize the tasks (which is against the common business goals dedicated to ensuring a successful task completion process independently of any assistance to users).

These categories of severity assessment levels (low, medium, high) are commonly used in usability testing practice, and such categories correspond to the standard categorization of issue severity outlined in usability testing literature [3,18]. Thus, such classification of the usability issue severity helps prioritize remediation efforts and aligns with the evaluators’ judgment-driven assessment of the impact on user performance.

#### 5.4.4. SUS Score Calculation

After processing all feedback data collected during the usability testing, in the last phase of the data analysis process, the SUS score calculation was performed. The individual SUS scores obtained through the SUS questionnaire were used to calculate the overall perceived usability of both the Figma prototype and the live web system.

## 6. Results

This section presents the key findings of the usability testing and SUS evaluation conducted on the Figma prototype and the live web system. The results are structured to provide insights into the root cause of usability issues, frequency and distribution of usability issues, overlap between issue occurrences identified in both test environments, and SUS scores calculated for each system. Together, the findings presented in this section provide a comprehensive comparison of the two test environments, highlighting their individual contributions and their complementary role in identifying and addressing usability challenges. The presented results of the statistical analyses determine how well Figma prototype testing predicts the real-world usability of an analyzed web-based system, and enable the identification of any additional usability issues that might emerge in the testing of web systems running in the live environment.

### 6.1. Repeated Usability Issue Occurrences

**Figure 8:**
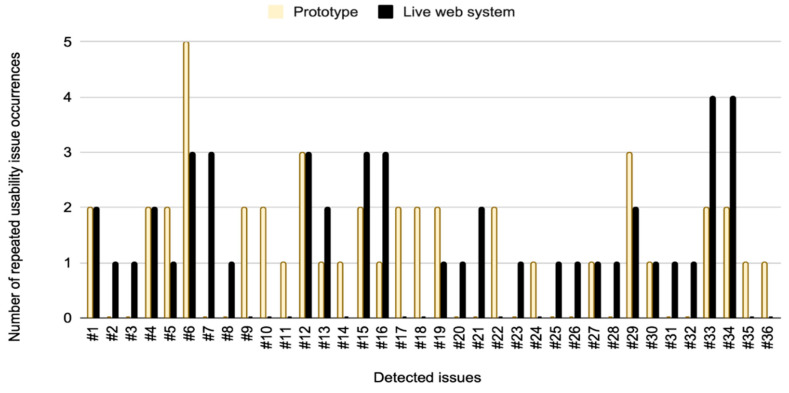
Number of repeated usability issue occurrences.

To provide a deeper qualitative insight into detected usability issues and to address the specific nature of the encountered problems, Table 7 presents a detailed characterization of detected usability issues based on severity level and test environment occurrence. According to Table 7, a total of 36 distinct usability issues were detected throughout the performed usability tests. While Table 7 contextualizes these issues by analyzing the root causes and describes specific test participants’ interaction barriers found in both the prototype and live web test environment, Figure 8 illustrates the frequency of issue occurrence.

Therefore, Figure 8 presents the results of a statistical analysis of the number of repeated occurrences for each specific usability issue. The data in Figure 8 and the description of each issue in Table 7 show that certain issues were exclusive to specific test environments. For example, issue #7 was not detected during the testing of the Figma web system prototype. (Figure 8) While some issues occurred only once, the majority of detected issues appeared multiple times across different user sessions. Notably, in the Figma prototype testing, issue ##6 had the highest frequency, appearing five times, compared to three occurrences of issues in the live web system testing (Figure 8). Among 36 unique usability issues identified across both test environments, 24 issues were detected in the Figma web system prototype and 26 in the live web system. Overall, live web testing uncovered 72.2% of all usability issues, while testing on the Figma prototype identified 66.7% of all usability issues detected in this study. The higher number of issues observed in the live system is attributed to production bugs, which could not appear in the Figma prototype.

### 6.2. Distribution of Issues by Severity

**Figure 9:**
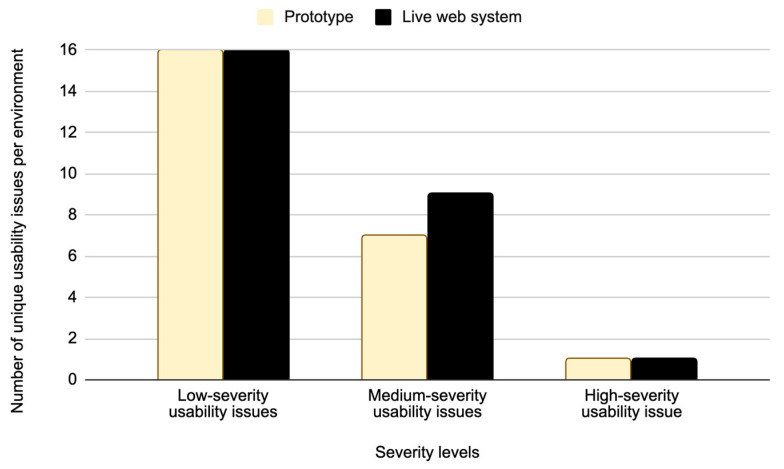
Distribution of the total number of unique usability issue occurrences for Figma prototype and live web system test environments based on severity levels.

Figure 9 presents the distribution of unique usability issues identified in the Figma prototype and the live web system, categorized by severity levels. As shown in Figure 9, a total of 50 unique usability issues were detected in both testing environments. The 16 unique low-severity usability issues were detected in both the Figma prototype and the live web system. Additionally, seven unique medium-severity usability issues were identified in the Figma prototype and nine in the live web system (Figure 9). Both environments revealed one unique high-severity usability issue. Results presented in Figure 9 show that there is a significant correlation in the distribution of detected usability issues when comparing the live web system and the Figma prototype. A possible reason for this high correlation is the advanced level of refinement of the Figma prototype and its high degree of alignment with the live web system.

**Figure 10:**
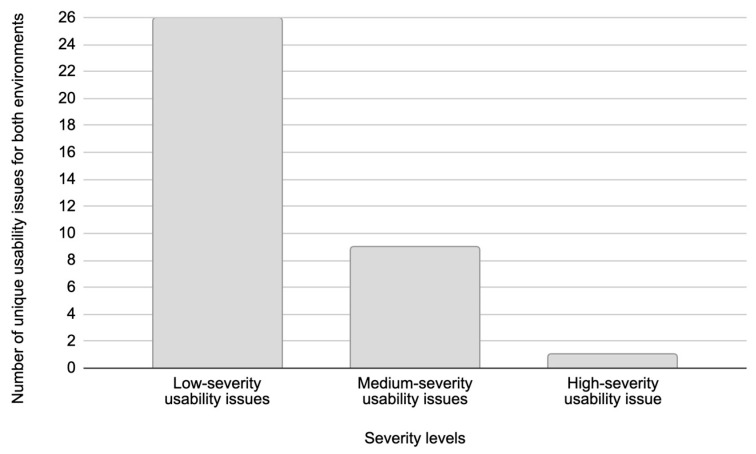
Distribution of unique usability issues for both test environments based on severity levels.

Figure 10 presents the total number of distinct usability issues grouped by severity levels, which are identified across both test environments. In this study, a unique usability issue represents a specific problem encountered during testing, which is counted only once regardless of how many times test participants experienced it. Figure 10, however, presents the total number of usability issues recorded across all usability testing sessions. This recorded total number of usability issues reflects how many issues were detected in total during testing. Given that usability testing was conducted with five test participants for the Figma prototype and five test participants for the live web system, this data captures all instances where usability issues were identified, even if the same issue occurred multiple times.

**Figure 11:**
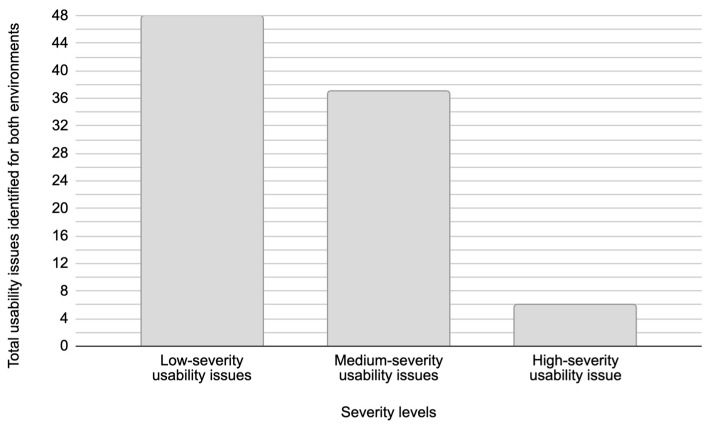
Distribution of the total number of usability issues identified during testing for both environments based on severity levels.

According to Figure 10, low-severity usability issues accounted for 26 unique issues, while Figure 11 shows that these issues appeared a total of 48 times across both environments during testing. This corresponds to an average issue occurrence of approximately 1.85 times per issue. Medium-severity usability issues consisted of nine unique issues, which were observed 37 times in total during testing (Figure 11), with an average issue occurrence frequency of approximately 4.11 times per issue. High-severity usability issues were identified as a single unique issue in both environments, appearing a total of six times during testing, with two issue occurrences in the Figma prototype and four in the live web system. This resulted in an average issue occurrence of 6.00 times per issue during testing.

These findings suggest that the frequency of usability issues is closely related to their severity. Low-severity usability issues often cause minor inconveniences or brief hesitation in system usage and are largely influenced by a user’s subjective perception, prior experience, and personal expectations. This individual variability makes these issues less consistent across users. In contrast, medium- and high-severity usability issues present significant barriers to task completion or entirely prevent users from completing tasks, respectively. These types of issues are typically more objective in nature and less dependent on individual user behavior, leading to their more frequent and consistent occurrence. This distinction underscores the importance of prioritizing the resolution of medium- and high-severity usability issues, as they have a greater impact on overall system usability and user satisfaction compared to low-severity usability issues.

### 6.3. Identifying Overlapping Usability Issues

**Figure 12:**
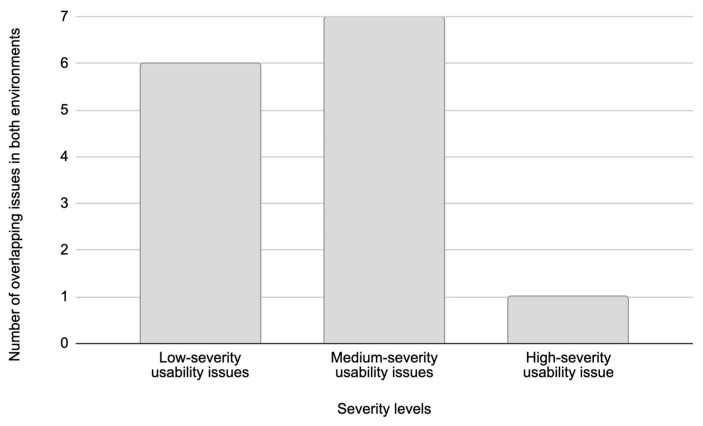
Distribution of the total number of overlapping usability issues in both test environments based on severity levels.

Based on the results presented in Figure 8 and Figure 9, the analysis is further focused on identifying overlapping issues and differences in usability issues detected across the two test environments. An overlapping issue is defined as a usability issue that was identified in both the Figma prototype and the live web system. The overlapping issues indicate the level of consistency in user experience challenges across testing environments. Figure 12 presents the distribution of the total number of overlapping usability issues in both test environments based on severity levels.

Low-severity usability issues accounted for the highest number of unique issues, totaling 26 usability issues across both environments (Figure 10). However, only six of these usability issues overlapped between the Figma prototype and the live web system (Figure 12). In contrast, medium-severity usability issues showed a much higher degree of overlap, where out of nine unique medium-severity usability issues, seven usability issues were common to both test environments. The remaining two medium-severity usability issues that were exclusive to the live web system were identified as production-specific bugs that could not appear in the Figma prototype.

The limited overlap in low-severity usability issues can be attributed to their subjective nature. As explained in Section 5.2, low-severity usability issues typically cause minor inconveniences that are highly influenced by individual user perceptions, prior user experience, and personal user expectations. This variability in causes of low-severity usability issues leads to a diverse range of low-severity usability issues, many of which are unique to each user and, therefore, less likely to appear consistently across different testing environments.

In contrast, medium-severity usability issues are generally more objective and subject to design flaws or interaction barriers that affect most users similarly, regardless of their digital literacy, prior experience with the service and brand, or familiarity with similar products. This consistency in user response explains the higher overlap of medium-severity usability issues between the Figma prototype and the live web system. Such issues tend to disrupt the user journey in predictable ways, making them more detectable during both prototype and live system testing.

In the performed usability testing, high-severity usability issues were rare but critical, with a single unique issue identified in both environments (Figure 12).

The substantial overlap of medium- and high-severity issues between the Figma prototype and the live web system indicates that prototype-phase usability testing reliably identifies the same high-impact issues later observed in production on live systems. Treating these overlaps as leading issue indicators enables timely intervention during prototyping, which can enable reducing late-stage development rework, accelerating product development, and lowering usability risk at digital service release. Early remediation thus supports a more robust, higher-quality digital service release with fewer design flaws. Thus, this finding underscores that severe usability issues can often be detected early in the design phase, allowing for timely intervention and resolution before full product deployment.

Specifically, the qualitative analysis presented in Table 7 reveals that issues related to financial information clarity were identical across both environments. For instance, the high-severity issue regarding the “Total” price label (Issue #33) and the medium-severity issue related to the ribbon pricing display (Issue #5) were detected in both the Figma prototype and the live web system test environments. This confirms that such design deficiencies are digital platform-agnostic and can be identified during the early prototyping phase.

Of 36 unique usability issues detected (Figure 9), in total, 14 different usability issues overlapped across both the Figma prototype and the live web system (Figure 12, Table 7). Of the remaining usability issues, 10 were unique to the Figma prototype, and 12 were exclusive to the live web system. These findings underscore the complementary roles of prototype and live system testing in the usability evaluation process. While prototyping effectively identifies a broad spectrum of usability issues during the early stages of development, testing on live systems uncovers additional challenges related to implementation and real-world user interactions.

### 6.4. SUS Results for Figma Prototype and Live-Web-System Testing

According to Table 1, all five participants who took part in the usability testing of the Figma prototype also completed the standard SUS questionnaire. Similarly, all five participants involved in the usability testing of the live web system also completed the SUS questionnaire, resulting in a total of ten participants who participated in an in-person SUS evaluation (Table 1).

The SUS score for each participant from both (Figma prototype and live web system) groups was calculated using the previously described procedure presented in the Section 4.3.1. SUS Questionnaire Score Statistics. For each group (Figma prototype and live web system), the mean value representing the SUS score was calculated based on the relation (1). Additionally, the standard deviation (SD) and the 95% confidence interval (CI 95%) were determined according to relations (2) and (3), respectively.

**Figure 13:**
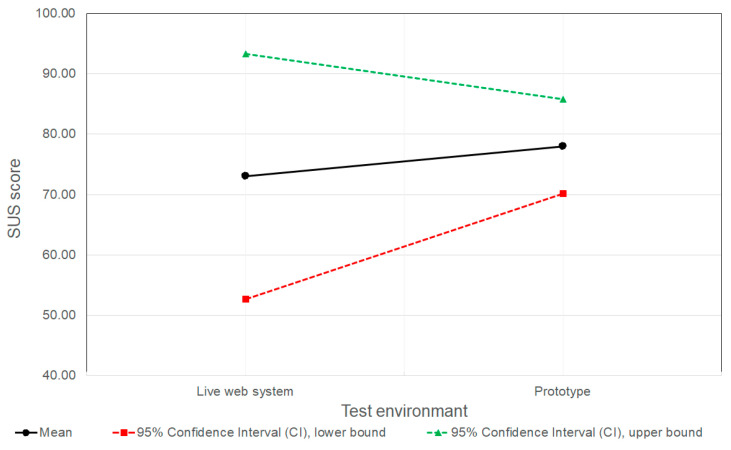
Statistical analyses of the SUS score for the live web system and the web system prototyped in Figma.

Figure 13 presents the statistical analysis of the SUS score for the live web system and the web system prototyped in Figma. The SUS score collected for the five participants who participated in the Figma prototype SUS evaluation (Table 1) had a mean SUS score of 78.00 (N= 5; SD = 8.9), with a CI 95% in the range [70.19, 85.8]. The SUS score for the live-web-system SUS evaluation, in which five test participants participated, was 73.00 (N = 5; SD = 23.14). Due to the significantly higher standard deviation observed in this group, the calculated CI 95% is in the wide range of [52.71, 93.29].

The mean SUS scores for the Figma prototype and the live web system are comparable, with 78.00 for the Figma prototype compared to 73.00 for the live web system. However, as shown in Figure 13, the confidence interval width for the live web system (40.6) is wider than that for the Figma prototype (15.6).

A narrower confidence interval and lower standard deviation indicate greater consistency in the responses of participants who tested the Figma prototype compared to the live web system. This greater consistency for the Figma prototype can be attributed to the context of testing, in terms that participants were explicitly informed that they were interacting with a web system prototype. This may have led them to attribute any misunderstandings or obstacles to the incomplete or unfinished nature of the system. Consequently, participants may have approached the task with certain expectations, resulting in more uniform perceptions of usability. In contrast, participants testing the live web system likely had a clearer sense of what to expect in terms of system performance and functionality, given that the live web system represents the final, implemented version of the digital product. As a result, variations in responses could stem from users being more critical of the live system when their expectations were not fully met, while others may have responded more positively due to features and functionalities that exceeded their expectations.

The results suggest that conducting SUS evaluations on prototypes can be a valuable predictor of the perceived usability of the final product. The small difference in mean SUS scores between the Figma prototype and the live web system suggests that SUS results obtained during prototype testing can reliably indicate the expected user satisfaction and perceived usability of the system, once it is fully developed and goes live in production. While the mean scores remained relatively close, the discrepancy in variance suggests that prototype testing reliably indicates the general usability trend, although it may underestimate the variability of user reactions compared to the real-world setting. Thus, the findings presented in this work underscore the value of incorporating SUS evaluations into the digital-product design phase. This enables assessing how users are likely to perceive the system’s usability, and provides valuable insights into overall user satisfaction early in the development process.

### 6.5. Discussion on Iterative Testing on Prototype and Live Digital Systems

Performing usability testing iteratively on a digital-web-system prototype (e.g., developed in the Figma application) and the live web system is valuable because it lets digital-product development teams separate design issues from implementation issues, and make faster decisions regarding the development of a better digital product. More specifically, testing both versions of the digital product iteratively helps distinguish design problems from technical problems.

For example, if users struggle in both the prototype and the live system testing, the issue is likely conceptual or UX-related (layout, wording, flow, etc.). If users struggle only in the live system testing, the issue is likely technical (performance, bugs, responsiveness, accessibility, etc.), and if users struggle only in the prototype, the prototype may be misleading, incomplete, or over-simplified. This distinction is hard to make when testing in only one test environment (prototype or live system).

Iterative testing also enables validating that the implemented system matches the intended design, since design intent often changes subtly during development, and by testing a prototype representing what designers meant to build and a live system representing what was actually built, it is possible to uncover missing interactions, altered flows, copy or hierarchy changes, edge cases dropped during development, etc. This is especially important for complex workflows (such as implementations of checkout, onboarding, forms, etc.).

Also, iterative prototype and live digital-system testing reduces the risks of implementing costly changes that eventually might be unnecessary. When teams rely only on live-system testing, fixes are expensive, and changes may require rework across code, quality assurance, and deployment. When teams rely only on prototype testing, they risk validating something that will not work well in practice. Thus, iterative testing allows them to safely explore improvements in the prototype, confirm the real-world impact in the live system, and avoid investing in changes that will not actually improve usability.

### 6.6. Discussion on Research Limitations and Future Work

The research presented in this study has limitations that should be considered when interpreting the results. Since the study was conducted on a single software (digital) system (e.g., Figma web prototyped and live-web system) that includes a user journey related to the telecom contract extension web service, the results of the study should be interpreted as exploratory and indicative in nature. Accordingly, the research presented in this work is positioned as a study aimed at evaluating the methodological approach for comparing digital system usability between a developed Figma interactive web prototype and its counterpart live web system. Therefore, this study tends to serve as a basis for future studies involving multiple services of the digital systems and a broader number of test participants.

Therefore, future research will expand the comparative methodology introduced in this work by including additional product development stages, multiple digital services, and larger and more diverse participant groups. Further research should also examine the influence of different sensing device characteristics (screen size, operating system version, browser type, hardware capability) on usability issues’ manifestation more systematically. Additionally, the strong predictive alignment observed among web prototype and web live SUS scores suggests exploring predictive modeling approaches, in which prototype-phase SUS results and usability metrics could be used to estimate expected live-system usability performance. Finally, broader validation of the newly introduced Croatian SUS translation across additional user groups and system types would contribute to its standardization and wider applicability in regional UX research and practice.

## 7. Conclusions

Due to a lack of research related to comparing usability test and System Usability Scale (SUS) results on real, operating digital systems and their equivalent prototypes, this paper presents a comparative analysis of usability testing and SUS evaluation performed on an identical live web system and its counterpart prototype developed in the Figma application. Unlike prior research works typical for live systems that are focused on single-phase UX evaluations without comparing the usability test results for the same user journey across multiple product development environments, analyses performed in this paper examine the usability of the same user journey on versatile mobile sensing devices for two test environments of the equivalent digital system. This analyses enable quantification of test usability issues overlap based on their severity and occurrence frequency, and comparison of SUS results across both test environments for different mobile sensing devices. The comparison was based on data collected from participants in moderated (attended) usability testing sessions. To evaluate user satisfaction and perceived usability, SUS results were derived using a standardized 10-item questionnaire, and a statistical analysis was performed on both usability testing outcomes and SUS data.

The results of this study demonstrate that prototype testing is highly effective for detecting a wide range of usability issues early in the digital product development phase. Most medium- and high-severity usability issues identified on different sensing mobile devices in the live web system were also detected during prototype testing, underscoring the ability of prototypes to replicate critical user interactions and predict potential obstacles in the realization of the digital product. The usability testing results also confirm no impact of differences in sensing mobile devices used in usability testing on the testing results. This reinforces the value of addressing usability issues during the digital product design phase, where changes are less costly and easier to implement.

However, testing of live digital systems remains indispensable for identifying additional challenges specific to real-world implementations. Factors such as data entry processes, backend system performance, production bugs, and real-time error handling often introduce new usability issues that are not observable in prototypes. These findings highlight the unique complexities of digital live systems, which prototypes, despite their high fidelity, cannot fully replicate.

Additionally, this study demonstrated that the SUS evaluation, when applied to prototype testing, can effectively anticipate the perceived usability of its counterpart digital system once it is developed and deployed. While variations in user feedback between the prototype and live system were observed, the overall consistency of SUS scores suggests that evaluations conducted during the design phase can provide a reliable indication of user satisfaction with the final product. This makes SUS evaluations a valuable addition to early digital-product usability testing, offering insights that can guide design decisions towards improving the satisfaction of users with respect to digital-product usability.

In summary, this research highlights that prototype testing effectively identifies key usability issues early in the digital-product development phase and provides a strong foundation for assessing perceived usability. Additionally, live-system usability testing uncovers implementation-specific challenges that impact real-world interactions. Therefore, future work will be dedicated to further analyses of usability testing results for the case of testing digital products that integrate prototype and live-digital-system test phases, in order to ensure digital-product designers and developers have a more robust and comprehensive evaluation of digital-product usability, ultimately reducing costs associated with late-stage revisions.

## Figures and Tables

**Table 1:** Distribution of participants and tests across test environment types.

Test Environment Type	Number of In-Person (Attended) Test Participants (Usability Testing and SUS Evaluation)
Figma prototype	5
Live web system	5
Both environments (total)	10

**Table 2:** Items of the SUS questionnaire and translation into the Slovenian and Croatian languages.

Original SUS Questionnaire (In the English Language) [11]	Slovenian Translation [29]	Croatian Translation (Created for the Presented Research)
I think that I would like to use this system frequently. I found the system unnecessarily complex I thought the system was easy to use. I think that I would need the support of a technical person to be able to use this system. I found the various functions in this system were well integrated. I thought there was too much inconsistency in this system. I would imagine that most people would learn to use this system very quickly. I found the system very awkward to use. I felt very confident using the system. I needed to learn a lot of things before I could get going with this system.	Menim, da bi ta sistem rad pogosto uporabljal. Sistem se mi je zdel po nepotrebnem zapleten. Sistem se mi je zdel enostaven za uporabo. Menim, da bi za uporabo tega sistema potreboval pomoč tehnika. Različne funkcije tega sistema so se mi zdele dobro povezane v smiselno celoto. Sistem se mi je zdel preveč nekonsistenten. Menim, da bi se večina uporabnikov zelo hitro naučila uporabljati ta sistem. Sistem se mi je zdel neroden za uporabo. Pri uporabi sistema sem bil zelo suveren. Preden sem osvojil uporabo tega sistema, sem se moral naučiti veliko stvari.	Mislim da je ovo sustav koji bih često koristio. Sustav mi se činio nepotrebno kompleksnim. Sustav mi se činio jednostavnim za korištenje Mislim da bi mi za korištenje ovog sustava bila potrebna podrška tehničke osobe. Različite funkcionalnosti ovog sustava čine mi se dobro povezane u smislenu cjelinu. Sustav mi se činio previše nedosljednim. Mislim da bi većina ljudi vrlo brzo svladala korištenje ovog sustava. Sustav mi se činio nespretnim za korištenje. Bio/la sam vrlo siguran/a u sebe prilikom korištenja sustava. Trebalo mi je mnogo učenja da bih se mogao snaći u ovome sustavu.

**Table 3:** Standardized moderator script and observation guidelines for Steps 1 to 4.

**Step 1: Initial email notification and login screen** **Question: Scenario:** You received an email from your telecom. Open it; what is your next step? **Question:** Comment on the screen content. **Question:** Login: What is your next step? **Observation**: Is the required input information clear to the participant? Do they know where to find it? **Note:** *If the participant decides to look for the login info on the bill, provide the sample bill*. **Question:** Now, imagine this is your bill. What do you do next?
**Step 2: Internet and TV package selection** **Question:** How do you interpret the internet options and their differences? **Question:** Which one would you normally choose? **Question:** If you wanted to learn more details about a specific option, what would you do? **Question: Mesh device:** Do you know what this is? Where would you look for information about it? **Question: TV:** How do you interpret the options? Which matches your preference? **Question:** If you wanted to check if your desired package includes a specific channel, how would you do that? **Question** (if clicking the channel list link): What do you expect to happen?
**Step 3: TV viewing options selection** **Question:** Comment on the available TV viewing methods. How do you perceive the different options available to you? **Question** (if unclear): How would you find the missing information? **Question:** Do you think you can install/activate the TV viewing application on your current television? **Question** (if unsure): How would you verify if your TV is compatible?
**Step 4: Device selection** **Question:** Comment on the presented options. **Question: Scenario:** You are interested in the e-scooter. What would you do if you wanted to buy it? **Question:** Estimate how many devices you can select. **Question** (after selection): If you wanted to cancel this purchase, how would you do that? **Question** (after removal): **Scenario:** You decide to buy it after all. How do you proceed? **Question:** How do you move to the next step?

**Table 4:** Standardized moderator script and observation guidelines for Steps 5 to 8.

**Step 5: Additional services selection** **Question:** Comment on the presented options. **Question: Scenario:** You are interested in Netflix. Do you have enough information to subscribe? What is missing? How would you find it? **Question: Task:** Let’s say you want the Standard Netflix package. How would you select it? **Question: Home Assistance:** How do you interpret the Home Assistance option? What do you think you get by subscribing? **Question:** Give an example of a situation where you might use this service. Are there any details missing? Where would you look for them?
**Step 6: User information update** - **Question:** Review the presented options and share your thoughts. - **Question:** Why do you think your mobile number is required for Netflix? - **Question:** How do you interpret the Bill option?
**Step 7: Order preview** - **Question:** Comment on what you see. - **Note:** *Remind the participant that the numbers/prices may not be real.* - **Question:** What do you think you need to do next? - **Question:** What do you expect to happen when you do that?
**Step 8: Success page** **Question:** Comment on what you see. **Question:** Is everything clear to you? **Question:** What do you think you need to do next? What do you expect to happen?

**Table 5:** Information about participants selected for usability testing.

Test Environment	Participant Number	Gender (M—Male/F—Female)	Level of Education	Age Group (Years)	Period of Previous Telecom Service Usage (Years)
Figma prototype	1	M	Secondary Education	45–55	14
Figma prototype	2	F	Master’s Degree	36–44	15
Figma prototype	7	F	Bachelor’s Degree	25–35	5
Figma prototype	8	M	Bachelor’s Degree	36–44	13
Figma prototype	10	M	Bachelor’s Degree	36–44	8
Live web system	3	M	Master’s Degree	36–44	15
Live web system	4	F	Bachelor’s Degree	25–35	15
Live web system	5	F	Secondary Education	36–44	6
Live web system	6	M	Bachelor’s Degree	45–55	9
Live web system	9	F	Bachelor’s Degree	36–44	10

**Table 6:** Examples of some of the detected issues categorized by severity and context.

Issue Severity	Issue Description
Low	The basket ribbon’s expand indicator is overlooked; the user does not realize the basket can be expanded and misses details.
Low	During the TV package selection, the channel list modal is not alphabetized, so the user must scroll a long, unsorted list to check if a desired channel is included in the package.
Medium	In the purchase basket, the “Basic Netflix” plan does not display the discounted price for the first 6 months; the user sees only the standard price and cannot verify that the promotion is applied.
Medium	During the login step, the “Customer ID” field lacks context; the user does not know which ID is needed and where to find it.
High	The ambiguous “Total” label conflates the monthly fee with one-time charges; the user misreads the cost breakdown and indicates they would abandon the purchase at this step.

**Table 7:** Overview of identified usability issues in both test environments.

Issue Number and Description	Severity	Environment
**#1 Email notification—‘Grab a scooter’ copy.** Test participants searching for “Renew contract” were confused by the “Grab a scooter” option. They failed to link this promotional hook with the service extension, which causes hesitation.	Low	Prototype and live web
**#2 Email notification—Hero image click.** Test participants expected the hero image to lead to the renewal flow. Instead, they were redirected to an unrelated page, causing disorientation.	Low	Live web -only
**#3 Email—Footer icons.** Test participants mistook decorative footer icons for buttons. They attempted to click them, expecting shortcuts to the service renewal page.	Low	Live web -only
**#4 Login—Credential confusion.** The label “Customer Code” lacks context and leads test participants to insert their password or contract number. The interface did not indicate that this customer code is found on the monthly bill.	Medium	Prototype and live web
**#5 Login—Ribbon pricing.** Test participants questioned the origin and validity of prices displayed in the top-aligned basket ribbon prior to any interaction or login; it was unclear if the figures were generic or personalized.	Medium	Prototype and live web
**#6 Package selection—Basic vs. additional TV.** Test participants failed to grasp that all packages share the same core channels, but differ in specific add-ons.	Medium	Prototype and live web
**#7 Package selection—TV channel filter bug.** The mode for viewing channels by TV package contained a broken filter that disappeared or functioned incorrectly.	Medium	Live web -only
**#8 Package selection—TV channel list sorting.** The lack of alphabetical sorting or search functionality made it difficult for test participants to check if and in which packages specific TV channels were available.	Low	Live web -only
**#9 Package selection—Home Box Office (HBO) without TV service.** Uncertainty about whether HBO required an active TV service led test participants to skip the offer.	Low	Prototype -only
**#10 Package selection—HBO cancellation.** Test participants could not verify if the HBO add-on allowed flexible cancellation. Fear of a long-term commitment caused them to decline the offer.	Low	Prototype -only
**#11 Package selection—Missing Netflix.** Promoting Netflix in the email led participants to expect it as an included option during TV package selection. Its absence in this step created diferences between expectations and reality.	Low	Prototype -only
**#12 Package selection—Mesh device inclusion.** The price tag for an “Additional Mesh device” displayed immediately below the package details caused participants to question if the primary included unit was truly free or subject to the shown fee.	Medium	Prototype and live web
**#13 Package selection—Internet speed data.** Raw numerical speed values failed to convey actual performance benefits. Test participants struggled to interpret the metrics, often confusing connection speed with data volume.	Low	Prototype and live web
**#14 Package selection—Contract toggle.** The 24-month obligation toggle was unclear. Vague wording prevented test participants from understanding the consequences of disabling it.	Low	Prototype -only
**#15 TV viewing—Option distinction.** Users were unable to easily distinguish between the different TV viewing options or identify their current setup. The lack of a clear benefits explanation made it difficult to determine the desired choice.	Medium	Prototype and live web
**#16 TV viewing—Philips TV support.** The offered service compatibility list relied on operating systems (e.g., Android TV) rather than brand names. Test participants with Philips TVs did not realize their devices were supported.	Medium	Prototype and live web
**#17 TV viewing—Android box details.** Tech-savvy test participants expressed dissatisfaction with the lack of detailed hardware specifications for the Android box in the info mode, preventing them from assessing device quality.	Low	Prototype -only
**#18 TV viewing—Conditional Access Module (CAM) card info.** Test participants searched for but could not find information regarding the CAM card option that enables decrypting and viewing encrypted subscription channels.	Low	Prototype -only
**#19 Device selection—Multiple devices.** It was unclear to test participants whether they could select one or multiple devices at the discounted price.	Medium	Prototype and live web
**#20 Device selection—State inconsistency.** Clicking “Show more” after selecting a device triggered an invalid interface state. The selected unit remained visible in the full list (marked as “Added”) instead of being separated.	Low	Live web -only
**#21 Device selection—Back navigation.** Clicking “Back” from a selected device returned test participants to the list, but the device remained incorrectly marked as “Added” (functional error).	Low	Live web -only
**#22 Additional services—Netflix contract.** Test participants were uncertain if the Netflix subscription was tied to the main contract’s 24-month term.	Low	Prototype -only
**#23 Additional services—Netflix modal device info.** The comparison table omitted the number of supported devices (concurrent streams) per tier.	Low	Live web -only
**#24 Additional services—HBO grouping.** Test participants found the separation of HBO and Netflix illogical, expecting these comparable streaming services to be grouped together, rather than split across different flow steps.	Low	Prototype -only
**#25 Additional services—Netflix discount.** Test participants were unsure if the discount applied to all service plans, as the interface did not clarify that the offer was exclusive to the “Basic” service plan.	Low	Live web -only
**#26 Additional services—Netflix promo price.** The test participants’ (customer) basket failed to apply the 6-month promotional discount for the “Basic” Netflix service plan, incorrectly displaying the full monthly fee.	Medium	Live web -only
**#27 Additional services—“Without fee” label.** The test participants were unsure if this phrasing implied a completely free service or merely addtional charge (surcharge) exemption.	Low	Prototype and live web
**#28 Additional services—Netflix modal table.** Test participants struggled to interpret the information points presented within the Netflix comparison table.	Low	Live web -only
**#29 Additional services—Home Assistance scope.** Test participants incorrectly assumed this home assistant service was for telecom issues instead of general home repairs.	Low	Prototype and live web
**#30 Additional services—Home Assistance pricing.** Test participants were unable to interpret the pricing terms displayed in the info mode, failing to understand what the pricing figures represented.	Low	Prototype and live web
**#31 Test participant info—Validation persistence.** The validation error triggered by a missing phone number failed to clear after the test participant entered valid data.	Low	Live web -only
**#32 Test participant info—Data loss.** The entered email value failed to persist when the test particiant navigate back from the next step.	Low	Live web -only
**#33 Order preview—Monthly total.** The ambiguous label “Total” caused confusion about the price breakdown. Test participants failed to realize that it included the device installation, leading to order abandonment.	High	Prototype and live web
**#34 Order preview—Payment options.** The absence of a credit card as payement option caused test participants dissatisfaction.	Low	Prototype and live web
**#35 Success page—Information placement.** Test participants felt it was illogical for selected additional devices to be displayed below the promotional block for the operator’s mobile application, expecting them to be more prominent.	Low	Prototype -only
**#36 General—Basket ribbon.** Test participants missed the interactive element to expand the basket ribbon. The lack of a prominent visual cue prevented them from accessing their order summary.	Low	Prototype -only

## Data Availability

The original contributions presented in this study are included in the article. Further inquiries can be directed to the corresponding author.

## Abbreviations

The following abbreviations are used in this manuscript:

CTA	Concurrent think-aloud
CI	Confidence interval
CAM	Conditional Access Module
CSUQ	Computer System Usability Questionnaire
HCI	Human–computer interaction
HBO	Home Box Office
H-SUS	Hybrid System Usability Scale
IPTV	Internet Protocol Television
ISO	International Organization for Standardization Standard Organization
NPS	Net Promoter Score
P-SUS	Pictorial System Usability Scale
RTA	Retrospective think-aloud
QUIS	Questionnaire for User Interaction Satisfaction
SD	Standard deviation
SUS-SI	Slovene SUS version
SUS	System Usability Scale
TV	Television
UMUX	Usability metric for user experience
UEQ	User experience questionnaire
UEQ-S	User experience questionnaire—short
UX	User experience
